# Microbial Primer: Bacterial energy metabolism

**DOI:** 10.1099/mic.0.001694

**Published:** 2026-04-20

**Authors:** Qonita Afinanisa, Alexander Brooks, Iremide Sanyaolu, Ashwathi Valiyaparambil, Tim W. Overton

**Affiliations:** 1 School of Chemical Engineering and Institute of Microbiology and Infection, The University of Birmingham, Edgbaston, Birmingham, UK

**Keywords:** anaerobic metabolism, energy metabolism, fermentation, respiration

## Abstract

All cells need energy to perform their functions. This primer introduces energy metabolism in bacteria, with a focus on key pathways and examples from model organisms, while acknowledging that bacterial diversity means that species such as *Escherichia coli* are not always ‘typical’ in terms of energy metabolism. It finishes by looking at how metabolism underpins important bacterial behaviours.

## Why do bacteria need energy?

All cells need energy – indeed, the harvesting of energy is a fundamental requirement of life and a classifier of living things. Energy is needed for processes such as movement, chemical reactions (typically anabolic reactions that generate larger molecules from smaller building blocks) and the transport of molecules across membranes against a concentration gradient. Energy cannot be created or destroyed, only converted from one form to another; therefore, living organisms must harvest or capture energy from their environment. Bacteria also require carbon, a fundamental atomic component of biochemicals and, thereby, cells. Acquisition of energy and carbon is often linked.

## Where does energy come from?

Humans gain energy and carbon from the same source, the breakdown (catabolism) of organic molecules (the food we eat); we are therefore classed as *chemoheterotrophs*. Many bacteria do the same, for example, the model organisms *Escherichia coli* and *Bacillus subtilis*. However, we must acknowledge that bacteria are incredibly diverse, and many bacterial species can harvest energy from light (photosynthesis – *phototrophs*) or inorganic chemicals like iron or sulphur (*lithotrophs*). Likewise, many bacterial species ‘fix’ carbon from CO_2_, like green plants (*autotrophs*) [1] . Within ecosystems, phototrophs, lithotrophs and autotrophs are often referred to as ‘primary producers’, in that they capture energy and carbon from the environment and provide them to consumers (heterotrophs like humans and *E. coli*). For the remainder of this primer, we will consider energy metabolism in chemoheterotrophs, whilst acknowledging the broader diversity of bacterial energy and carbon metabolism.

## The basics: aerobic glucose metabolism

The preferred carbon and energy source for many bacteria (e.g. *E. coli*) is glucose – for this reason, many descriptions of energy metabolism start with glucose. In the presence of oxygen, glucose is completely oxidized (equation 1).


(1) $$C_{6}H_{12}O_{6}+6O_{2}\to 6CO_{2}+H_{2}O$$

This reaction liberates energy; oxidation of glucose in a chemistry laboratory would be called combustion and would release energy in the form of heat. In biological systems, the energy released is captured in different ways, most commonly in the form of chemical energy-carrier molecules, the most ubiquitous being ATP. ATP is hydrolysed to ADP with the loss of a phosphate group, releasing energy, which is used to drive work within the bacterium. Typically, in bioenergetics, this useful energy is stated as Gibbs free energy (G) and the release of energy as a negative change in G, Δ*G*. ATP is used as an energy carrier as the Δ*G* of hydrolysis of ATP to ADP and inorganic phosphate (P_i_) is highly negative. ATP can be thought of like a charged battery, whereas ADP can be compared to a discharged battery. Glucose oxidation is used to drive the production of ATP from ADP, thereby ‘recharging’ the energy store.

Metabolism of glucose also generates intermediate chemicals, required as building blocks for the synthesis of diverse biomolecules and new cells, and reducing power in the form of the electron carriers NAD and NADP. These reversibly associate with electrons and protons and are required to enable reduction in a range of enzymatic reactions, and for the final stages of respiration.

**Fig. 1.**
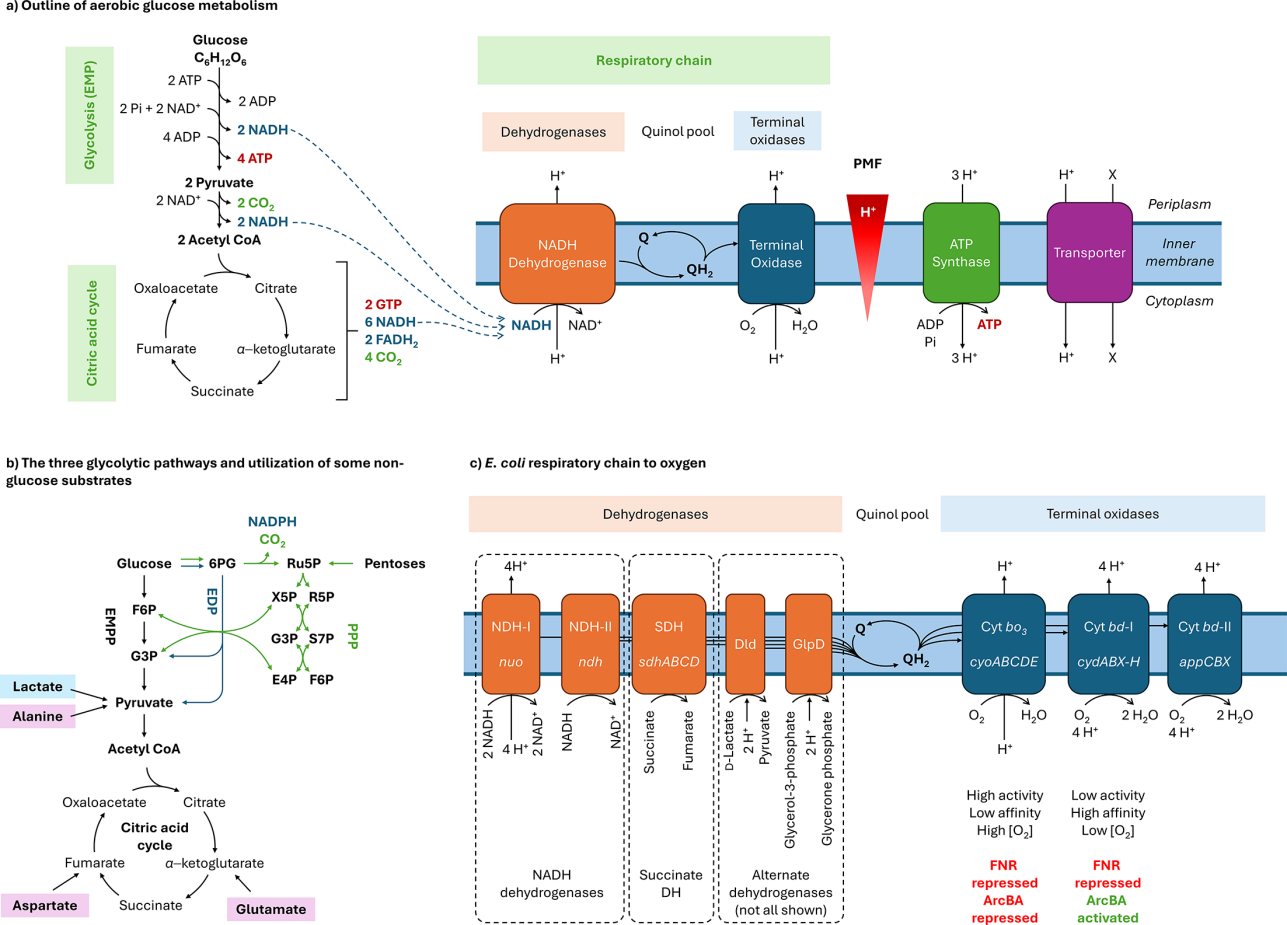
Summary of aerobic energy metabolism with a focus on *E. coli*. (**a**) Simplified outline of aerobic glucose oxidation. Summary of glycolysis (the EMPP), the citric acid cycle and a simplified respiratory chain. Not all intermediates are shown. The EMPP comprises ten reactions that convert glucose to two pyruvate molecules and yield two ATPs and two NADHs per glucose. It should be noted that two ATP molecules are initially consumed per molecule of glucose, with the final generation of four ATP molecules, resulting in an overall yield of two ATPs. The citric acid cycle oxidizes the carbon in pyruvate to CO_2_, yielding two GTPs per glucose plus reducing power in the form of NADH and FADH_2_. Reducing power from NADH and FADH_2_ is fed into the respiratory chain through dehydrogenases, which reduce the quinone (Q) pool to quinols (QH_2_). The reduced quinols then pass electrons to the terminal oxidases, which reduce terminal electron acceptors (O_2_ in the case of aerobic respiration). Protons are pumped across the inner membrane, generating the PMF, which is used to drive ATP synthesis as well as other functions, such as transporters. (**b**) Summary of sugar metabolic pathways. The three glycolytic pathways are the EMPP, EDP and PPP. Abbreviated intermediate names and the number of carbons in each molecule are: F6P (six carbons), G3P (three carbons), 6PG (six carbons), Ru5P (five carbons), X5P (five carbons), R5P (five carbons), S7P (seven carbons) and E4P (four carbons). Not all steps and intermediates are shown for clarity. (**c**) Outline of the aerobic respiratory chain in *E. coli*. Selected dehydrogenases are shown on the left (some alternative dehydrogenases are not shown for clarity), the quinol pool in the centre (comprising three quinols) and the three terminal oxidases on the right, summarizing their enzymatic features and aspects of regulation below. E4P, erythrose-4-phosphate; EDP, Entner–Doudoroff pathway; F6P, fructose-6-phosphate; G3P, glyceraldehyde-3-phosphate; GTP, guanosine triphosphate; 6 PG, 6-phosphogluconate; PPP, pentose phosphate pathway; R5P, ribose-5-phosphate; Ru5P, ribulose-5-phosphate; S7P, sedoheptulose 7-phosphate; SDH, succinate dehydrogenase; X5P, xylose-5-phosphate.

Glucose is oxidized via multiple reactions, which can be split into three stages: glycolysis, the citric acid cycle and the respiratory chain (Fig. 1a). Glycolysis converts glucose to pyruvate. There are three glycolytic pathways in *E. coli*, the most prevalent being the Embden–Meyerhof–Parnas pathway (EMPP), which comprises ten reactions and yields two ATP molecules and two NADH molecules per glucose. The pyruvate generated by glycolysis is then completely oxidized to carbon dioxide by the citric acid cycle (eight reactions, also referred to as the Krebs cycle), with release of energy in the form of guanosine triphosphate (comparable to ATP), and reducing power [NADH and flavin adenine dinucleotide (FADH_2_), the latter a protein-bound electron carrier]. Both glycolysis and the citric acid cycle occur in the bacterial cytoplasm.

The reducing power liberated by these first two stages then drives the respiratory chain, which occurs at the cell (inner) membrane; a model respiratory chain is shown in Fig. 1a. The electrons are transferred from NADH, through a series of membrane-spanning multi-enzyme complexes (the respiratory complexes), until they reduce oxygen to water. These enzymes transfer electrons through redox-active cofactors including haem (in cytochromes), flavin and iron–sulphur centres. Electrons pass from a negative to a positive redox potential, releasing energy at each step.

NADH is first oxidized to NAD^+^ by an NADH dehydrogenase, which transfers the electrons to quinols (Q), hydrophobic electron-carrier molecules localized within the lipid portion of the membrane. *Quinol* refers to the reduced molecule (QH_2_) and *quinone* to the oxidized form (Q). The quinols migrate laterally through the membrane, shuttling electrons to the terminal oxidase, which reduces oxygen to water. Oxygen is thereby the terminal electron acceptor. The energy released by electron transfer permits some respiratory complexes to pump protons outside the membrane, generating an electrochemical gradient, the proton motive force (PMF). A major role of the PMF is to provide energy to the ATP synthase enzyme, where protons pass back to the cytoplasm, driving ATP synthesis. This process is referred to as oxidative phosphorylation.

The PMF is also used to drive a range of other cellular functions at the membrane, such as the transport of molecules across the membrane against a concentration gradient by transporters, efflux of antimicrobial compounds by efflux pumps and rotation of the flagella permitting motility. The PMF is a component of the membrane potential, reviewed by Benarroch and Asally [2]. Overall, oxidation of one glucose molecule can yield up to 38 ATP molecules; however, this number decreases if the PMF is used to power other functions, or if carbon intermediates are channelled to other biomolecules in the bacterium.

## Adding complexity: metabolic diversity in carbon sources

Building on the simple scheme given above, most bacteria are able to utilize a range of substrates to support growth. To use a substrate, it has to enter the bacterium (usually via a transporter) and be chemically transformed to enter central carbon metabolism. The substrate range of different species therefore depends upon their genomic repertoire of transporters and enzymes.

There are three pathways for hexose (six-carbon sugar) utilization. We have introduced the EMPP above; many bacteria (although not *E. coli*) convert hexoses to pyruvate via an alternative glycolytic pathway, the Entner–Doudoroff pathway, comprising only five enzymatic steps. The third major pathway for monosaccharide utilization is the pentose phosphate pathway, interconverting sugars containing three to seven carbons, permitting diverse sugar utilization, and generating NADPH, an important source of reducing power for biosynthesis reactions.

Disaccharides and polysaccharides are typically broken down to monosaccharides before entering central carbon metabolism; for example, lactose is split by *β*-galactosidase (LacZ) in *E. coli* to glucose and galactose, which enter the EMPP. Other common substrates include organic acids and amino acids, which are typically converted to organic acids (a process called transamination) before entering central carbon metabolism. This is effectively the opposite of amino acid synthesis.

The range of substrates that a given species can utilize is usually a reflection of which molecules are available in its environment; many human pathogens are able to utilize lactate, which is found in many tissues. Many selective growth media permit the identification of bacteria based on the range of substrates they can use.

## Aerobic respiratory chain flexibility and diversity

The respiratory chain is slightly more complicated than introduced above, with multiple dehydrogenases feeding electrons to the quinol pool, driving multiple terminal oxidases. Different species also possess different respiratory chain architectures [3] and can use their respiratory chain flexibly, depending on growth conditions and oxygen concentrations.

In *E. coli* (Fig. 1c), there are two NADH dehydrogenases: NDH-I (encoded by the *nuo* operon), which is proton-pumping, and NDH-II (*ndh*), which does not pump protons. NDH-I is also referred to as Complex I, as it has homology to the human mitochondrial complex of the same name; respiration is thereby deeply conserved through evolution.

NDH-I and NDH-II pass electrons to three quinols: ubiquinol, menaquinol and demethylmenaquinone. Electrons are also passed to quinols by succinate dehydrogenase (*sdhABCD*), which catalyses the reduction of succinate to fumarate in the citric acid cycle, using FADH_2_ as an electron carrier. Other dehydrogenases, including GlpD and Dld, oxidize glycerol-3-phosphate and lactate, respectively.

Quinols pass electrons to one of three terminal oxidases, varying in activity and affinity. Cytochrome *bo* (*cyoABCDE*) is proton-pumping and high-throughput, but has low oxygen affinity, so it is used at high oxygen concentrations. Cytochrome *bd*-I (*cydABX-cydH*) has a higher affinity for oxygen, so it is used at low oxygen concentrations (microaerobic conditions). Expression of these operons is repressed by the oxygen-sensing transcription factor FNR and is differentially regulated by the two-component regulatory system ArcBA, which senses the redox state of the quinol pool, repressing *cyo* but activating *cyd*. This demonstrates that energy metabolism is carefully regulated at the level of transcription. The third terminal oxidase, cytochrome *bd*-II (*appCBX*), is less well characterized. The complexity of the *E. coli* respiratory chain is reviewed by Unden *et al*. [4].

*Pseudomonas aeruginosa* has a more complicated, branched aerobic respiratory chain, with three NADH dehydrogenases and several other dehydrogenases feeding electrons into the quinol pool. There are five terminal oxidases, two of which take electrons directly from the quinol pool, with the remaining three channelling electrons from quinol via two intermediate cytochromes (*bc*_1_ and *c*). This diversity allows *Pseudomonas* to live in a variety of environments with varied oxygen tensions. Furthermore, this species generates hydrogen cyanide, which inhibits most terminal oxidases; *P. aeruginosa* must therefore express a cyanide-insensitive terminal oxidase. Moving away from Gram-negative species, *Staphylococcus aureus* possesses two NADH dehydrogenases, both NDH-II type (there is no NDH-I/Complex I in this species), and two terminal oxidases (Qox and Cyd).

## Life without oxygen: anaerobic energy metabolism

Unlike humans, many bacterial species can survive in the absence of oxygen; facultative anaerobes (such as *E. coli*) are those that can grow without oxygen, whereas obligate anaerobes require oxygen-free conditions. Aerobically, the reducing power carried by NADH is used to reduce oxygen to water. In the absence of oxygen, bacteria must direct the reducing power to other end points. There are two approaches to this: fermentation, where the reducing power is used to directly reduce intermediates of central carbon metabolism, eliminating the respiratory chain; and anaerobic respiration, where electrons are used to reduce molecules other than oxygen (called alternative electron acceptors). Anaerobic metabolism varies widely between different species, both in terms of whether fermentation or respiration is preferred, and the range of substrates and electron acceptors.

**Fig. 2.**
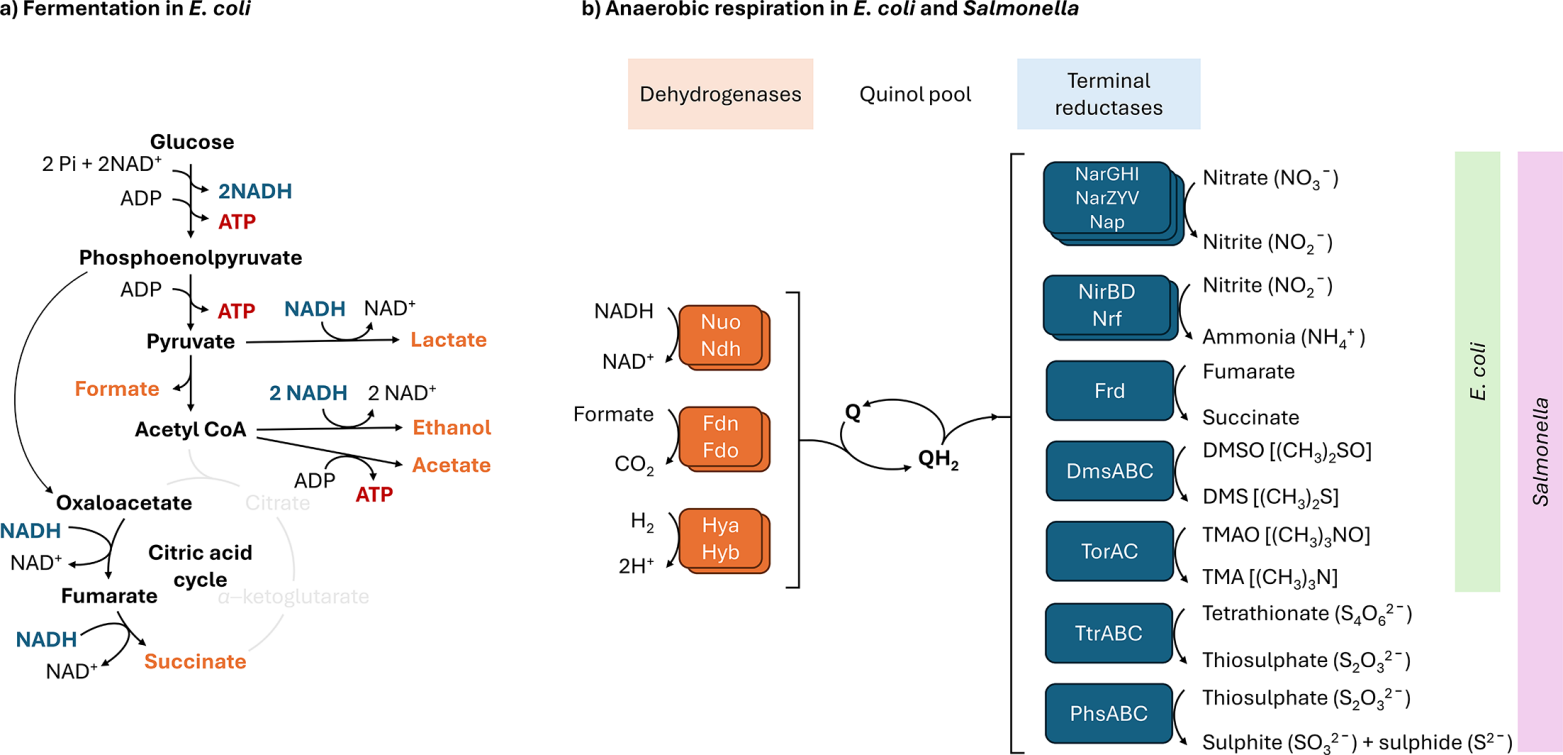
Aspects of anaerobic energy metabolism in *E. coli* and *Salmonella*. (**a**) Mixed-acid fermentation in *E. coli*. End products are shown in orange, indicating which pathways consume reducing power (NADH) and/or generate ATP. Conversion of acetyl CoA and oxaloacetate to succinate via the citric acid cycle does not occur under these conditions, as indicated by greyed-out steps. Not all steps and intermediates are shown for clarity. (**b**) Anaerobic respiration in *E. coli* and *Salmonella*. Relevant dehydrogenases are shown on the left, and terminal reductases on the right, each with their substrates and products. DMS, dimethyl sulphide; TMA, trimethylamine; TMAO, trimethylamine *N*-oxide.

1. *E. coli* can ferment glucose to a range of reduced end products, commonly referred to as mixed-acid fermentation (Fig. 2a). The generation of different end products utilizes different quantities of NADH and also generates different quantities of ATP, permitting flexibility depending on whether the bacterium requires NAD^+^ recycling or energy. It should also be noted that formate and hydrogen generated by fermentation can drive respiration; formate dehydrogenase and hydrogenase enzymes transfer electrons to the quinol pool (Fig. 2b).
2. *E. coli* can use five alternative electron acceptors: nitrate (NO_3_^−^), nitrite (NO_2_^−^), fumarate, DMSO and trimethylamine *N*-oxide (Fig. 2b). *Salmonella* adds tetrathionate and thiosulphate to this list. The terminal reductase enzymes catalysing these reactions are located at the inner membrane; their expression is regulated by the availability of oxygen (mediated by FNR and ArcBA) and by their relevant electron acceptors (e.g. the nitrate- and nitrite-sensing two-component regulatory systems NarXL and NarQP). Similar to terminal oxidases, there are multiple nitrate and nitrite reductases, with higher and lower affinity and activity. It should be noted that not all dehydrogenases can donate electrons to all terminal reductases; coupling is energetically constrained by the redox potential of each enzyme.

## Real-world implications of energy metabolism

Bacteria have evolved to live in different niches; energy metabolism reflects this, with each species being able to utilize the substrates and electron acceptors (oxygen or alternatives) present in each environment. Energy metabolism, therefore, has a massive impact on bacterial physiology and behaviour; it underlies many aspects of bacterial behaviour and survival.

### Impacts on physiology: life in the host

The ability to adapt to different host environments plays a critical role in both bacterial pathogenicity and commensalism. Many bacteria have evolved to utilize host-derived molecules; lactate is the end product of fermentation in human cells and can be utilized by many bacteria as a carbon and energy source. During infection, *Salmonella* hijacks host cell energy metabolism to power its own metabolism in favour of bacterial proliferation and dissemination. *Salmonella* species can infect diverse niches in their hosts and therefore can adapt to different host environments.

During colonization of the gut, *Salmonella* Typhimurium exploits the metabolism of both the host and the gastrointestinal microbiome by inducing inflammation [5]. This provides *Salmonella* with lactate and oxygen (both generated by host metabolism) and the alternative electron acceptors nitrate and tetrathionate, which are used in respiration of microbiota-derived 1,2-propanediol and ethanolamine as carbon sources. In addition, succinate produced by the gut microbiota can be catabolized by *Salmonella*, acting as a source of carbon to complete the citric acid cycle.

*Salmonella* Typhimurium can also survive inside human macrophages; the *Salmonella* pathogenicity islands encode effectors that reprogramme macrophage metabolism to provide carbon sources, including 2- and 3-phosphoglycerate and phosphoenolpyruvate [6]. There are many such examples of bacteria modulating human metabolism for their benefit.

### Impacts on physiology: biofilm formation

Most bacteria live not planktonically, but in biofilms, assemblies of bacteria enclosed within a self-produced extracellular polymeric substance (EPS) matrix. Many biofilms are problematic to humanity, for example, those causing infections. Nutrient and oxygen concentrations within biofilms vary spatially, not only due to limitation of diffusion through the EPS matrix but also due to utilization by bacteria within the biofilm, giving rise to varied microenvironments. Most naturally occurring biofilms comprise multiple species and strains, each potentially having their own preferred energy sources and electron acceptors, increasing the complexity further.

In oxygen-limited (anoxic) microenvironments, for example, deep in the biofilm, bacteria can use alternative electron acceptors. Previous work has shown that, in *in vivo* and *in vitro P. aeruginosa* biofilms, bacteria in the outer layers rapidly respire oxygen while the inner cells switch to nitrate reduction [7]. Unlike *E. coli*, *P. aeruginosa* reduces nitrate to N_2_, a stepwise process via NO_2_^−^, NO and N_2_O called denitrification. Interestingly, simultaneous oxygen and nitrate respiration can occur at the same depth within the biofilm. Oxygen inhibits the last step of denitrification, generating N_2_O as the end product. However, the ATP produced can still contribute to the energy demand across the varying oxygen gradient. This shows that cells can physiologically adapt to different micro-niches. An intermediate of denitrification, NO, can also induce biofilm dispersal, further amplifying the heterogeneity of biofilms.

New evidence suggests a more fundamental link between energy metabolism and biofilms. There is a growing body of literature showing that respiration can lead to formation or dispersal of biofilms (termed *respiration-induced biofilm formation* or *respiration-induced biofilm dispersal*) in a species- and strain-dependent manner (reviewed by Martín-Rodríguez [8]). This emerging link could offer novel routes to prevent formation or drive dispersal of biofilms, which is especially important given that biofilms are often resistant to antimicrobials.

### Impacts on physiology: bioprocesses

As well as posing problems to humanity, bacteria are also used in productive processes. For decades, *E. coli* has been used for production of industrially relevant recombinant proteins, such as the drug insulin. In these processes, there needs to be a balance between biomass generation and product formation, both requiring intermediates such as amino acids, energy and reducing power. Recombinant protein production is described as posing a metabolic burden on bacteria.

In such processes, *E. coli* is typically grown in bioreactors, usually to very high cell densities (optical densities at 650 nm >100, >40 g dry cell weight per litre) [9]. This poses a problem because high quantities of carbon source are needed to support such high biomass accumulation. However, supplementation of growth media with a high concentration of glucose would result in overflow metabolism, whereby glucose is fermented to acetate and other acids rather than being respired, even in the presence of sufficient oxygen. This is not only wasteful (reducing ATP yield) but also reduces culture pH, inhibiting growth. Industrial solutions to this problem include use of a fed-batch growth method, where glucose is added gradually through the process, use of glycerol as a carbon source (which does not generate as much acetate as glucose) and use of *E. coli* B strains such as BL21, which produce less acetate due to changes in metabolism.

Another example of useful microbes is lactic acid bacteria, a grouping of multiple bacterial species, including *Lactobacillus*, which are widely used in the dairy industry and as probiotics. Many *Lactobacillus* species primarily harvest energy through homolactic fermentation, a pathway that generates lactate as a sole end product. Surprisingly, many of these species possess an aerobic respiratory chain but are unable to synthesize haem cofactors and quinones [10]. Providing these bacteria with exogenous haem and menaquinone permits respiration and has been shown to improve growth in industrial settings.

Acid production is relevant to the use of *Lactobacillus* spp. in the dairy industry, transforming milk into yoghurt and preventing spoilage by competing microbes. Competition is also likely the natural function of lactate production; *Lactobacillus* spp. are human commensals and live in polymicrobial environments, such as the gut and vagina. In these environments, haem and menaquinone are available, supporting some respiratory growth.

## Conclusions

We can see that energy metabolism is diverse, is often complicated and is driven by the resources available to a given bacterial species in its natural habitat. It can therefore give clues to the habitat and overall lifestyle of the organisms. In particular, the study of the interplay between bacterial energy metabolism and host metabolic pathways is offering insights into pathogenesis, commensalism and complex interactions within the microbiota.

When growing bacteria in the laboratory, one should think about how growth conditions are allowing them to harvest energy. Indeed, given the diverse mechanisms of energy generation possessed by many species, one should consider how different carbon sources and electron acceptors are influencing behaviour, gene regulation and physiology.

Finally, it may be surprising to learn how little is known about energy metabolism in some species. Genome sequencing has allowed far greater understanding of the enzymes encoded by different species and strains, so predictions of preferred routes of energy metabolism can be made, but experimental confirmation is often required, especially where novel pathways are involved.

## Abbreviations

EMPP: Embden–Meyerhof–Parnas pathway
EPS: extracellular polymeric substance
FADH_2_: flavin adenine dinucleotide
G: Gibbs free energy
P_i_: inorganic phosphate
PMF: proton motive force
